# Mechanisms of Immunotoxicity: Stressors and Evaluators

**DOI:** 10.3390/ijms22158242

**Published:** 2021-07-31

**Authors:** Maroun Bou Zerdan, Sara Moussa, Ali Atoui, Hazem I. Assi

**Affiliations:** 1 Department of Internal Medicine, Naef K. Basile Cancer Institute, American University of Beirut Medical Center, 1107 2020 Beirut, Lebanon; marounzerdan@gmail.com (M.B.Z.); aa326@aub.edu.lb (A.A.); 2 Faculty of Medicine, University of Balamand, 1100 Beirut, Lebanon; sara.moussa@std.balamand.edu.lb

**Keywords:** immunotoxicity, microbiota, hypersensitivities, allergies, immune system, cigarette smoke, stressors, chemicals

## Abstract

The immune system defends the body against certain tumor cells and against foreign agents such as fungi, parasites, bacteria, and viruses. One of its main roles is to distinguish endogenous components from non-self-components. An unproperly functioning immune system is prone to primary immune deficiencies caused by either primary immune deficiencies such as genetic defects or secondary immune deficiencies such as physical, chemical, and in some instances, psychological stressors. In the manuscript, we will provide a brief overview of the immune system and immunotoxicology. We will also describe the biochemical mechanisms of immunotoxicants and how to evaluate immunotoxicity.

## 1. Introduction and Overview of the Immune System

### 1.1. Immune Cells and Their Development

The immune system orchestrates the body’s main defense against invading biologic agents including but not limited to bacteria, viruses, chemicals, and foreign tissues. Lymphocytes, neutrophils, macrophages, eosinophils, and basophils are the main players. These cells are produced at an increased rate during childhood, where such a blood draw in a child would reveal an average number of 3000/mm^3^ compared to 4500–11,000/mm^3^ in adults [1], and the development of the human immune system begins in the fetal period and reaches its maximum capacity around puberty [1]. A multipotent stem cell gives rise to either a myeloid stem cell or a lymphoid stem cell. Eosinophils, basophils, and neutrophils arise from myeloblasts through granulocytopoiesis. Myeloid stem cells also give rise to monoblasts, which become monocytes and later on macrophages through monocytpoiesis. Lymphoid stem cells give rise to B-cells, T-cells, and natural killer cells. Controlled by negative feedback, the immune system’s headquarters is in lymphatic tissues. The primary organs are the bone marrow where immune cell production and B-cell maturation take place and the thymus where T-cell maturation takes place. The secondary organs are the spleen, lymph nodes, tonsils, and Peyer’s patches. The secondary organs allow immune cells to interact with antigens. Some T-cells and B-cells undergo further differentiation and pick up various functions [1]. T-cell precursors in the bone marrow move to the cortex in the thymus to undergo positive selection and later on to the medulla where they undergo negative selection. The former is when T-cells expressing T-cell receptors capable of binding self-major histocompatibility complex (MHC) on cortical epithelial cells survive. The latter is when T-cells expressing T-cell receptors with high affinity undergo apoptosis or become regulatory T-cells. These cells later on become cytotoxic T-cells, T-helper cells, or T-suppressor cells. Cytotoxic cells destroy target cells in order to avoid the progression of a virus infection or cancerous growth. Cytokines dictate the cell-to-cell regulation of the immune system. How these lymphokines interact is summarized in Table 1.

### 1.2. Innate and Adaptive Immune Cells and Their Activities

The immune system is subdivided into two complementary functions. The first is called the innate immunity, which is made up of neutrophils, macrophages, dendritic cells, natural killer cells, complement, chemokines, physical epithelial barriers, and secreted enzymes [2,3,4]. The innate immunity is germline encoded and resistance persists through generations and does not change within an organism’s lifetime. The adaptive immunity’s components are the T-cells, B-cells, and antibodies, and it undergoes variation through V(D)J recombination during lymphocyte development. Microbial resistance here is not heritable. While the innate immunity’s response to pathogens is nonspecific and occurs rapidly with no memory response, the adaptive immunity is highly specific, is refined over time, develops over long periods, and a memory response is formed. Once a memory response is formed, subsequent exposure to a previously encountered antigen results in a stronger and quicker immune response.

The innate immunity functions through toll-like receptors, pattern recognition receptors that recognize pathogen-associated molecular patterns (PAMPs) and lead to the activation of NF-κB. Some of these PAMPs are lipopolysaccharides present on Gram-negative bacteria, flagellin, and nucleic acids.

On the other hand, the adaptive immune response is much more intricate. The adaptive immune response is split into humoral and cellular immunity. T-cell activation and B-cell activation, along with class switching, occurs. Specialized antigen presenting cells called dendritic cells take up antigens and migrate to the draining lymph node. Foreign antigens are presented on MHC II in dendritic cells and are recognized by T-cell receptors on CD4+ cells. Endogenous or cross-presented antigens are presented on MHC I to CD8+ cells [1]. After T-cell activation is done, T-cell proliferation and survival is achieved through a costimulatory signal via interaction of the B7 protein (CD80/86) on dendritic cell and CD28 on naïve T-cells. Concerning B-cells, the same steps just mentioned take place as well to activate a T-helper (CD4+) cell [1]. Antigens presented on T-cell receptors of activated T-helper cells interact with MHC II on B-cells. Next, CD40 receptors on B-cells bind the CD40 ligand on T-helper cells. Finally, T-helper cells secrete cytokines that determine immunoglobulin class switching of B-cells. B-cells are activated, undergo class switching and affinity maturation, and begin producing antibodies [1]. An excessive reaction is prevented from happening due to a negative feedback caused by a decrease in T-cells.

## 2. Immune Hypersensitivities and Autoimmune Disease

### 2.1. Autoimmune Diseases

Autoimmunity is more commonly seen in patients treated with recombinant cytokines. This further illustrates the leading role of cytokines in the immunopathological conditions. It is highly unlikely that a single cytokine is responsible for the pleiotropic manifestations of autoimmunity [5,6,7]. Autoantigens on different cell types are targeted by lymphocytes. There is often a balance between T-helper 1 cells (TH1) and T-helper 2 cells (TH2). The former is heavily involved in many autoimmune diseases. On the other hand, the latter secretes IL-4 and tends to act in an opposing effect. Most autoimmune diseases are TH1 driven except for a few such as ulcerative colitis and systemic lupus erythematosus. Some cytokines can either be therapeutic in some patients or induce an autoimmune disease in patients taking the recombinant form of some cytokines. These include but are not limited to IL-2, IFN-α, IFN-β, IFN-γ, and TNF-α [8]. Once these cytokines are overproduced, patients undergo clinical and observed immunological changes driven by an abnormal expression of MHC class II molecules. This abnormal expression is guided by IFN-γ, IL-1, and TNF-α. For example, when IFN-γ is expressed in high amounts, thyroid cells express MHC class II and act as antigen-presenting cells. The consequence of the latter is the production of antithyroid autoantibodies [8,9,10]. Finally, most drug-induced autoimmune reactions portray the same manifestations in terms of clinical and observed autoimmune diseases.

### 2.2. Hypersensitivity Reactions

Immune-mediated and nonimmune-mediated are the main broad divisions of hypersensitivity reactions. The former necessitates a prerequisite of sensitization, which sometimes can also be due to cross-allergenicity. While there are limited ways to prove previous contact to antigen, high clinical suspicion is sufficient most of the time. Molecules should be foreign and have a molecular weight of at least 5000 in order to be antigenic [11]. According to Landsteiner, haptens induce sensitization by binding strongly to a carrier protein. As a matter of fact, some intermediate metabolites seem to play a role as haptens. The most recent examples of immune-mediated hypersensitivity are cutaneous eruptions with carbamazepine [12], skin lesions induced by metamizole [13], and others [14]. Here, it is worth noting that whatever the sensitizing potential of a given antigen or xenobiotic, not everyone is expected to develop an immune-mediated reaction. There are some risk factors related to the patient such as age, gender, atopy, genetic predispositions, environment, and socio-economic status. Having a younger age, being female, being exposed to maternal smoking which increases atopy, and living in an industrialized country are a few examples. Other risk factors are related to the substance such as the chemical structure, route of exposure, and exposure regimen.

The four types of hypersensitivity reactions are easily distinguishable. Type I is known to be anaphylactic and atopic and it has two phases. The first phase is immediate and occurs within minutes. In this phase, the antigen crosslinks the preformed IgE on presensitized mast cells, which leads to immediate degranulation and release of histamine, a vasoactive amine, and tryptase, as a marker of mast cell activation. The second phase is known to be late and is characterized by chemokines, which attract inflammatory cells and other mediators such as leukotrienes from mast cells. The end result is inflammation and tissue damage. Example of type I reactions are allergic asthma and anaphylaxis [15,16]. The second type of hypersensitivity is known to be antibody mediated. Here, antibodies bind to cell-surface antigens and lead to cellular destruction, inflammation, and/or dysfunction. Some examples of cellular destruction are autoimmune-hemolytic anemia, immune thrombocytopenia, transfusion, and hemolytic diseases of the newborn. In the previously mentioned examples, the cell is coated by antibodies, leading to either phagocytosis and/or activation of a complement system or antibody-dependent cellular cytotoxicity. In cases of inflammation such as in Goodpasture syndrome and rheumatic fever, the binding of antibodies to cell surfaces leads to the activation of a complement system and Fc receptor-mediated inflammation. In cases of cellular dysfunction, antibodies bind to cell surface receptors and lead to either an abnormal blockade or activation of downstream processes. Some examples are myasthenia gravis, Graves’ disease, and pemphigus vulgaris [17,18,19,20]. Type III hypersensitivity is characterized by immune complex deposition and complement activation [21,22,23]. Serum sickness, Arthus reaction, systemic lupus erythematosus, and poststreptococcal glomerulonephritis are all typical examples. Finally, type IV hypersensitivity reactions can occur via two mechanisms, each involving T-cells but no antibodies. The first occurs through direct cell cytotoxicity where CD8+ cytotoxic cells kill targeted cells, and the second mechanism involves CD4+ T-cells, which recognize antigens and release inflammation-inducing cytokines [24,25,26,27,28,29].

Nonimmune-mediated reactions, previously known as pseudoallergy or nonallergic hypersensitivity, constitute an important part of hypersensitive reactions. Three main criteria should be fulfilled in order to avoid confusion when defining what is a nonallergic hypersensitivity reaction [30]. The first is that anaphylactic-like adverse events can develop in patients after first contact. The second is the involvement of the same vasoacting pro-inflammatory mediators. The third, which is debatable, is the ability to distinguish between idiosyncrasy and pseudoallergy. It is recommended to disregard adverse reactions that develop in patients with a predisposing genetic defect [31]. For example, acute hemolysis, which occurs in patients who have enzyme glucose-6-phosphatase dehydrogenase deficiency, is considered an idiosyncratic reaction. In that sense, an idiosyncratic always leads to an immunological injury without the overt involvement of reactive metabolites [32]. In some cases, a pseudoallergic reaction such as Hoigné’s syndrome, first described by Batchelor in 1951, can be mistaken for an allergic reaction even though there is no release of mediators or presence of genetic predisposition [33].

The mechanisms of peudoallergic reactions have not been fully understood [33,34], but some mechanisms have been explored. Direct histamine release, activation of the complement system, and the inhibition of kinin degradation are plausible explanations. Histamine release is seen with general intravenous anesthetics [35,36], morphine derivatives [37], and vancomycin [38]. Polyoxyethylated castor oil [39] and hydrosoluble radiological contrast agents [40] along with rituximab [41] are some of the examples where the complement is evidenced to be involved.

## 3. Mucosal Immunity, the Microbiome and Food Allergies

### 3.1. Mucosal Immunity

The mucosa stretches over a large area of the human body, spanning from the oral, ocular, and ear cavities to the respiratory, digestive, and genitourinary tracts [42]. This large surface is used by most pathogens as a main route of entry to the body whether by inhalation, ingestion, or sexual contact [43]. Therefore, it is crucial for the mucosa’s immunologic barrier to protect the host from potential external invasion. This ability of the immune system to mount an effective response relies heavily on the location of effector cells and their speed at recognizing foreign antigens [44]. Other factors may also be associated such as physical barriers, the ability of the lumen to digest foreign antigens after selectively binding to their sites, and different T cell subpopulations that modulate and regulate the immune response [45]. In fact, the absence of these regulatory mechanisms may result in increasing the pathologic burden and the development of infections and inflammation [46]. For instance, dysregulation of the oral epithelium’s barrier makes it prone to environmental threats, leading to a cascade of events possibly culminating in oral cancer [46]. On the other hand, repeated exposure to an antigen leads to a mechanism of immune unresponsiveness known as “tolerance” [47]. To put it differently, for example, in food allergy, the immune system fails to achieve this state of unresponsiveness to one or more foods that leads to the activation of effector T cells against food antigens instead of regulatory T cells [47]. Moreover, each T cell subtype is activated by a different range of molecules. TGF-β and high levels of CD103+ dendritic cells are required for the activation of regulatory T cells. These dendritic cells express two enzymes: retinal dehydrogenase,. which converts retinal to retinoic acid, as well as indoleamine 2,3-dioxygenase, which has immunosuppressive properties. However, if the immune system comes across an intolerable antigen or an adjuvant such as bacterial adjuvant toxins, CD103− CD11c+ dendritic cells promote effector T cells to mount an appropriate response and neutralize the antigen [48]. To sum up, mucosal immunity encloses local tissue based innate responses as well as systemic adaptive events, intrinsic defenses, and highly conserved cell cytoprotective responses [49].

### 3.2. Microbiota

From an immunological point of view, microorganisms have traditionally been viewed as pathogens to be eliminated by the immune system. However, several microbes normally inhabit the body and are referred to as “microbiota” [50]. In fact, this collaborative relationship between the host and the healthy microbiota does not interfere with the host’s function to fight off any invasive pathogen [50]. It has been shown that the intestinal microbiota is composed of approximately 17 families, 50 genera, and more than 1000 species of bacteria [51]. This composition is affected by stress, medications, diet, and age. For example, as one ages, Bacteroidetes and Firmicutes become prevalent, whereas Actinobacteria and Proteobacteria become less represented [51]. Changes in bacterial number and composition lead to a “dysbiotic microbiota”, which whether a cause or sequela, exacerbates the pathology of the disease and the treatment needed to restore a healthy symbiotic microbiota [52]. For instance, in inflammatory bowel syndrome, patients have fewer Lactobacilli and Bifidobacteria and increased numbers of aerobic bacteria compared to anaerobes [53]. On the other hand, the fetal gastrointestinal tract is known to be sterile; however, the human’s microbiota is developed later by exposure to microbes. The earliest exposure occurs at birth with the passage through the birth canal, followed by the exposure to maternal milk later [51]. The latter contains IgA, which restricts immune activation, as well as oligosaccharides that promote the growth of special microorganisms in the microbiota such as Bifidobacterium [54]. This newly developed microbiota has clear mutual benefits with the host: the host ensures the environment and growth factors necessary for bacterial proliferation, and bacteria undergo fermentation to generate butyrate, for example, a short chain fatty acid, which is used as a main source of energy for the gut’s epithelial cells [54]. Other short chain fatty acids have also been shown to play an important role in regulating tight junction proteins, which in turn lead to the preservation of the integrity of the epithelial barrier [55]. Furthermore, parts of the microorganism itself such as bacterial lipopolysaccharide (LPS) may act directly on the gut by initiating a toll-like receptor 4 (TLR4) mediated series of events, triggering signaling pathways such as nuclear factor kappa b (NF-κB) and mitogen-activated protein kinase (MAPK), culminating in inflammation [56]. Thus, the tricky part of the host-microbial homeostasis is to mount an appropriate response to maintain a healthy gut while at the same time preventing unregulated immune responses against the beneficial microbiota [56,57]. Bearing all that in mind, one can conclude that the microbiota can be seen as a “forgotten organ” that interacts with and benefits its host on several levels, achieving a mutualistic symbiotic relationship.

In recent years, there has been a growing interest in the role of the microbiome and vitamin D in diseased and normal states. Some of these diseases include but are not limited to infectious states such as COVID-19, cardiovascular, metabolic, autoimmune, and malignancies [58,59,60,61]. High levels of vitamin D or dysregulated levels often lead to the maturation inhibition of dendritic cells, which increases the risk of allergic sensitization. Vitamin D deficiency and dysbiosis can also inhibit the development of T-helper 1 responses [62]. Vitamin D is also involved in stimulating the production of components of the innate immune system such as pattern recognition receptors, anti-microbial peptides, and cytokines [63]. The microbiota profile can be altered by an autophagy gene, ATG16L1, which is controlled by intestinal epithelial vitamin D receptor (VDR) [64]. Furthermore, a heterogenous group of innate lymphoid cells found in mucosal cells maintain intestinal hemostasis. Preliminary studies by Chen et al. found that the VDR knockout mice had more IL-22-producing innate lymphoid cells and more anti-bacterial peptides when compared to wild type mice [65,66].

Vitamin D has also been shown to affect T lymphocytes. First of all, vitamin D has been shown to favor T-cell function and differentiation. Vitamin D has been shown to stimulate T-helper 1 cells, T-helper 2 cells, and T-helper 17 cells, all of which control immune tolerance [67]. Cantorna et al. compared vitamin-D sufficient mice (VitD+) to vitamin D deficient mice (VitD-). The former had higher levels of FoxP2+ and ROR RORγt/FoxP3+ Treg cells in the colon [68]. Finally, the relationship between vitamin D and B lymphocytes is limited to studies that found that 1,25(OH)2D3 appears to regulate Breg cells and stimulate IL-10 production [58].

### 3.3. Food Allergies

In recent years, special attention has been given to food allergies since an accurate diagnosis and a proper management plan are crucial to prevent anaphylaxis. It is estimated that in 2007, approximately three million children under 18 years old (3.9%) reported having food allergies, recording an 18% increase from 1997, with most of them being at two to four times at increased risk of having other allergies or asthma [69]. One can talk about food allergies when the immune system mounts an adverse response against a dietary protein associated with various signs and symptoms subsiding with elimination of the offending allergen from the diet [70]. It may affect nearly any system including the gastrointestinal and respiratory tracts, skin, and cardiovascular system [70]. As for the prognosis, it is good since most children outgrow their allergies with age. However, children with high concentrations of IgE, also known as atopy or predisposition, have persistent allergies. Other allergies, once present, are also considered lifelong such as allergies for peanuts and shellfish [71]. On the other hand, some organ specific diseases or food intolerances may sometimes be mistaken for food allergy. Such examples include the chronic inflammatory intestinal disease, Celiac disease, in which gluten triggers an immune response [48]. However, a true food allergy can be IgE mediated, non-IgE mediated, or mixed.

IgE mediated, also known as immediate type hypersensitivity or type I reaction, is typically of rapid onset with symptoms appearing clinically within minutes to few hours after ingestion [72]. It usually occurs in sensitized individuals, those with detectable food specific IgEm whom the mechanism of tolerance is lost, which leads to the perception of certain benign food antigens as pathogens by the body [72]. Class I allergens cause sensitization in the gastrointestinal tract where they can conserve their structure despite the physical movement, enzymes, heat, and acid, whereas class II allergens are respiratory tract related [48]. However, class II, which are often fruit or vegetable allergies, do not directly cause sensitization, but rather induce their effect through cross reactivity, which renders re-exposure to the allergen unnecessary [48].

Upon first exposure, antigen presenting cells of the intestinal lamina propria, especially dendritic cells, capture the food allergen, which will be internalized and then degraded [73]. The resulting allergenic fragments will be presented to naïve CD4+ T cells by the major histocompatibility complex class-II (MHC-II). This, along with IL-4, will drive the differentiation of CD4+ T cells into Th2 cells producing cytokines such as IL-4 and IL-13, which alongside the interaction of CD40 on B cells and the CD40-ligand on Th2, are capable of changing the B cells’ production of antibody to IgE [74]. Other cytokines like IL-4, IL-5, IL-6, IL-9, IL-10, and IL-13 are also produced by Th2 cells and are responsible for mediating some clinical symptoms by causing the induction of serum IgE levels and eosinophils [75]. Recently, researchers have investigated the role of IL-17 in allergic sensitization. The results showed that IL-17A is involved in inflammation and IL-17E induces Th2 cytokine production and eosinophils [76]. On the other hand, the Th1 subtype also contributes by producing interferon-γ, which helps in maintaining the chronicity of the allergic inflammation in conjugation with the listed Th2 cytokines [77]. After class switching, IgE immunoglobulin attaches to FcεR1 of the mast cells or basophil cells. Once mast cells are activated, degranulation occurs within minutes, releasing cytokines, chemokines, proteases, leukotrienes, and bioactive polyamines, each with a specific role [78]. The most important mediator released is histamine, which causes muscular constriction, vasodilation, and increases vascular permeability [48]. Other main metabolites synthesized by mast cells are arachidonic acid derivatives. PGD2 induces eosinophil infiltration and smooth muscle contraction. The latter is also a function of LTC4 in addition to mucus production and increasing vascular permeability, whereas LTB4 functions as a chemoattractant for neutrophils, eosinophils, and T cells [48]. Upon secondary exposure, the allergen-IgE-FcεR1 complex forms, triggering a non-intrinsic tyrosine kinase pathway. PLCγ and PI3K signaling molecules induce DAG and IP3, causing Ca++ release from the endoplasmic reticulum, ultimately leading to mast cell degranulation [79].

Non IgE-mediated disease, a type IV hypersensitivity or delayed type hypersensitivity, is typically chronic and may be more difficult than IgE-mediated disease to control with food avoidance alone [72]. It mainly encompasses diseases of the gastrointestinal tract such as food protein-induced enterocolitis syndrome (FPIES), food protein-induced allergic proctocolitis (FPIAP), and food protein-induced enteropathy (FPE), celiac disease, and cow’s milk-induced iron deficiency anemia [80]. The most common allergens triggering this type of food allergy are cow’s milk, soy, and cereals. In addition, eggs, vegetables and fruits, and poultry are common in young children, whereas fish, shellfish, and mushroom allergies are more prevalent in older age groups [48]. To date, no confirmatory noninvasive tests are available to diagnose these disorders. Therefore, the diagnosis is purely clinical and is confirmed if the symptoms subside upon food avoidance [81]. Clinical symptoms can range from emesis and dehydration in FPIES to diarrhea and malabsorption in FPE and blood streaked mucosy stools in AP [81]. As for the prognosis of such disorders, the majority of infantile non-IgE-GI-FAs have the best prognosis. However, in some of the affected patients, the manifestations are severe and lead to shock in certain forms of FPIES or FPE. On the other hand, in older children and adults, these disorders mimic inflammatory bowel disease [82]. The exact mechanisms underlying the pathology of this type of food allergy are still relatively poorly understood, but the fact that IgE antibodies are only produced locally instead of systematically suggests that local mucosal IgE might be involved in the pathophysiology [83]. A few studies that have investigated the underlying pathology of food protein-induced enterocolitis syndrome (FPIES) showed some evidence of T cell proliferation upon stimulation with certain food allergens, which led to the thought of FPIES being T-cell mediated, but more research needs to be done [83,84]. Chung et al. highlighted the vital role of intestinal IFN-ɤ in villous injury and showed that patients with FPIES had an imbalance between intestinal TNF-α levels and TGF-β [84,85]. Furthermore, they proved that food allergens stimulate these proinflammatory cytokines, which increase the intestinal permeability and cause fluid shifts that mediate the local intestinal inflammation that occurs in non-IgE mediated food allergies [85]. There is an urgent need to better characterize the pathophysiology of non-IgE-GI-FAs to better diagnose relying on biomarkers and to develop new possible treatment strategies [82].

Only one reference mentioned that food allergens or immune cells often interact with toxicants and toxins, which may not be food allergens by themselves, to induce food allergies [48]. Mycotoxins, the secondary metabolites of fungi, are the perfect example. Aspergillus flavus and aspergillus parasiticus produce aflatoxins that are extremely potent metabolites [86]. Aflatoxins, specifically aflatoxin B1 (AFB1), can induce toxicity by being teratogenic, mutagenic, carcinogenic, and immunosuppressive, affecting both innate and adaptive immunity [86]. Several studies have shown that AFB1 inhibited the proliferation of T and B lymphocytes and the phagocytic functions of natural killer cells and macrophages, rendering the individual more susceptible to infections [87]. On the other hand, insecticides, herbicides, fungicides, and fertilizers used nowadays in agriculture impose a great risk of immunotoxicity since they are present in raw and processed food as well as in the soil, water, and air [48]. Phillips et al. cohort’s study showed that pesticides caused an elevation in pro-inflammatory cytokines and neuropeptides, which suggests a generalized state of neurogenic induced inflammation with stimulation of the humoral immune system as proven by the increased amounts of IL-5 and the presence of clinical symptoms of allergy [88]. Moreover, another study was conducted to further investigate the role of pesticides during the prenatal and early postnatal periods. Duramad et al. proved a possible correlation between pesticides and the changes in T cell cytokine profiles, which resulted in several pediatric diseases ranging from allergic disorders and leukemia to respiratory illnesses and diabetes [89]. Finally, industrial exposure to fertilizers rich in ammonia increased the prevalence of respiratory symptoms including allergic asthma among factory workers [48]. However, no evidence exists to prove the association of phosphorus fertilizers with allergies [48].

## 4. Molecular Immunotoxicology of Environmental Stressors

### 4.1. Physical Stressors

Multiple studies are being conducted to demonstrate the role of ultraviolet (UV) radiation in suppressing the immune system. The process leading to this suppression is intricate, starting with skin chromophores absorbing UV photons, which cause an upregulation in T and B regulatory cells while inhibiting effector and memory T cell activation, culminating with local and systemic changes in immune mediators [90]. These systemic changes occur indirectly via skin produced mediators rather than directly, since UV is not capable of penetrating below skin level [48]. Platelet activating factors and alarmins are major mediators produced by keratinocytes, Langerhans cells, mast cells, and NKT cells, which induce immunosuppression after UV radiation exposure [91]. In fact, mast cell migration is a crucial step in the pathway of UV immune effects. This migration is stimulated the increased production of platelet activating factor by Langerhans cells, which activates its receptor on mast cells [48]. Keratinocytes also produce several immunosuppressive mediators such as TNF-α, IL-10, *cis*-urocanic acid, and prostaglandin E2 (PGE2) [48]. In addition, UV also exerts its downregulation of the immune system by activating bioactive lipids, alarmins, and IL-33 [91]. Wang et al.’s study on immunized mice that received UV irradiation showed that a CD4+ regulatory T cells cytokine, IL-10, inhibited Th cell activation and by that, antibody responses [92]. Their results further demonstrated that antigen specific IL-10 producing regulatory T cells dampened Th2 immunity, which could later be a base for asthma treatment investigations [92].

UV, especially the mid-wave range (UVB), is categorized as a complete carcinogen. This can be explained by its full ability to cause skin cancer without the help of other factors. However, co-factors can accelerate the onset of cancer or increase the chance of having multiple cancers [93]. In 1940, it was proven that UV absorption by DNA paralleled mutagenicity. In fact, years later, studies showed that UV radiation is highly genotoxic and capable of messing up the DNA repair mechanism, leading to mutagenesis and cell death [94]. It was subsequently found that UVC and UVB radiation exhibit their toxicity by inducing cyclobutane pyrimidine dimers (CPD) between adjacent pyrimidine bases in DNA strands [95]. Another UV induced dimer at di-pyrimidine sites is the 6 ± 4 photoproduct (6 ± 4 PP) [96]. Furthermore, following UV exposure, a dysregulation of keratinocytes and mast cells’ production of cytokines, mainly IL-10 and TNF-α, was observed, which plays a key role in the pathogenesis of skin cancer [97]. Regulatory T cells, precisely Ag-specific “suppressor” T cells, also appear to be involved in UV-induced immune suppression. CD4+CD25+CTLA-4+ cells that secrete IL-10 and CD4+ T cells that share similar markers with NK cells constitute a perfect example of these T cells [48].

Numerous studies have been performed to come up with preventative measures against UV exposure. For example, nicotinamide is a water-soluble vitamin B3 derivative that counteracts UV radiation’s inhibition of glycolysis and the reduction of bodily ATP, thus preventing UV-induced oxidative damage and energy crisis [98]. By doing that, nicotinamide favors DNA repair while attenuating UV’s immunosuppression [98]. In addition, many polyphenols, or phytochemicals, most of which are dietary supplements, have also exhibited potential skin photoprotective effects. For example, green tea polyphenol can reverse DNA damage by antagonizing the effects of UV on cyclobutane pyrimidine dimers, which is a main trigger of immunosuppression [48]. Yarovaya et al. explored the role of the grape seed extract GSE. They proved that at a concentration as high as 25 μg/mL, it increased dermal fibroblast viability and protected against UVA damage [99]. Furthermore, their results showed that when the extract is introduced to a sunscreen’s mixture, it has the capability to increase the UV filter absorption in the cream, offering a better protection from solar radiation [99].

### 4.2. Chemical Stressors

#### 4.2.1. Metals

Our understanding of the relationship between metals and the immune system continues to evolve. Observed effects include immunosuppression, immune stimulation, contact and pulmonary hypersensitivity, and autoimmunity [100]. Several studies chose arsenic (As) because it has been associated with various cancers and numerous other pathologies. In fact, people are at daily risk of exposure to it through the ingestion of contaminated food and water in countries such as Taiwan, Bangladesh, India, Chile, and the United States or through inhalation in agricultural and industrial settings [101]. A 2013 review showed that arsenic alters B and T lymphocyte functions as well as macrophage function, affecting both innate and humoral immunity [102]. Exposure to this metal induces oxidative stress, inflammation, and apoptosis, which render the host immunocompromised and susceptible to infections, cancers, and lung diseases [102]. The skin is a primary target organ for chronic arsenic toxicity. In fact, its effects range from lesions known as arsenical keratosis to squamous cell carcinoma in situ of the skin known as Bowen disease [103]. Chronic exposure can also cause a spectrum of liver pathologies such as hepatocellular carcinoma, angiosarcoma, cirrhosis, and hepatoportal sclerosis [103].

Beryllium (Be) and nickel (Ni) are the perfect examples of metals that cause hypersensitivities. Individuals that are exposed occupationally or non-occupationally to beryllium dust or fumes are at high risk of developing a non-caseating granulomatous inflammation that leads to chronic beryllium disease, which principally affects the lungs, lymphatics, and skin [104]. This resulting disease, which is caused by CD4+ T cells, occurs more in genetically susceptible persons whose adaptive immune responses, are mainly mediated through single nucleotide polymorphisms in HLA-DP and, to a lesser extent, HLA-DR [104]. On the other hand, high levels of nickel can inhibit the development of immune organs by extensively inducing apoptosis and inflammation [105]. These mechanisms are activated through toll like TL4-mediated nuclear factor-κB (NF-κB) and signal transduction cascades mitogen-activated protein kinase (MAPK) pathways [105].

Numerous studies have investigated the effects of lead (Pb), especially in children. This metal is capable of preferentially inducing Th2 and M2 macrophages and altering both humoral and cellular immune responses [106]. Cadmium (Cd) and mercury (Hg) also have immunomodulatory effects. Daum et al. showed that both CdCl_2_ and HgCl_2_ inhibit RNA, DNA, and antibody synthesis, thus exerting early, inhibitory effects on B-cell activation, influencing the host’s ability to mount an effective immune response [107]. Moreover, mercury as well as gold and iron can expose cryptic epitopes on self-proteins, tricking the immune system to perceive them as new peptides after associating with MHCII [48]. To put it differently, such metals can change the normal cleavage of self-proteins or bind to self-peptides, generating a new epitope for MHC presentation [48].

In sum, the impact of many metals on immunity is due to their influences of oxidative or anti-oxidative mechanisms involving inflammation and apoptosis [108].

#### 4.2.2. Cigarette Smoke

It has been shown that cigarette smoke affects macrophage function and mitochondria activity, potentially leading to the exacerbation of certain infections and re-activation of latent M. tuberculosis [109]. The toxicity of cigarette smoke lies mainly in two components. First, acrolein inhibits the activation of c-Jun, shutting down the macrophage responses and favoring the alkylation of Cys-61 and Arg-307 in the DNA binding region, leading to the inhibition of the NF-κB1 pathway [48]. The second element is 2,3,7,8-tetrachlorodibenzo-*p*-dioxin (TCDD), which is also the most toxic of the group of structurally related compounds known as halogenated aromatic hydrocarbons [110]. Some investigators have suggested that epithelial cells within the thymus are the endpoint for TCDD, while others have proposed that thymic atrophy is rather due to a direct effect on thymocytes, either by apoptosis or by altering the development of progenitor cells [111]. Regardless of the way to the endpoint, TCDD can mess with the adaptive immune responses including delayed-type hypersensitivity, cytotoxic T lymphocyte activity, and T cell dependent antibody responses, thus downregulating both cellular and humoral immunity [110]. Moreover, TCDD induced toxicity appears to be dose-dependent, with perinatal exposure resulting in more severe immunosuppression than exposure during adult life [112].

**Figure 1:**
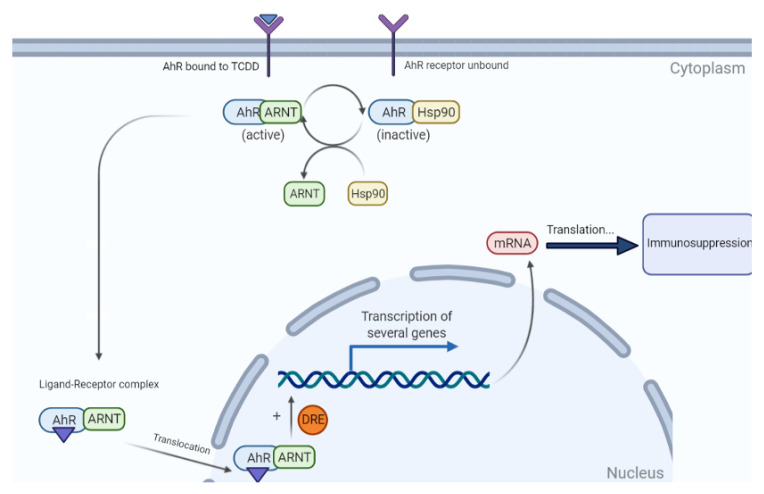
How the aryl hydrocarbon receptor functions.

Most, if not all, of the effects caused by TCDD and other aromatic hydrocarbons are known to be mediated by AhR (aryl hydrocarbon receptor or dioxin receptor). This receptor has a high binding affinity to TCDD and functions, similarly to the steroid receptor, as a ligand-activated transcription factor [113]. In the absence of a ligand, AhR remains in the cytosol bound by several chaperone proteins such as Hsp90 [114]. However, the binding of TCDD to AhR in the cell’s cytoplasm in a stereospecific manner stimulates the switching of AhR’s partner molecule from Hsp90 to ARNT, known as the AhR nuclear transporter [113]. The formed ligand-receptor complex is then translocated into the nucleus to initiate transcription. The trigger is the binding of the complex to a specific DNA enhancer sequence, called dioxin-responsive elements (DRE), with high affinity [110]. The result of this process is an enhanced expression of structural genes such as CYP1A1 in hepatocytes, which encodes the mRNA necessary to produce the enzyme cytochrome P450-1A1 and other products that regulate the differentiation and proliferation of cells [110]. However, the exact mechanism by which TCDD exerts its immunosuppressive role remains largely unknown [115]. Figure 1 summarizes the previously mentioned process. Thus, researchers need to shed more light on the immunotoxic effects of cigarette smoke and establish a clearer understanding of the mechanism of TCDD’s immunosuppression.

#### 4.2.3. Pesticides and Other Organic Compounds

Toxic substances can disrupt the immune system at many levels and pesticides are no exception. A pesticide, whether insecticide, herbicide, or fungicide, is any substance used to kill, repel, or control specific plants or animals [116]. It executes its immunological effect by inducing thymic atrophy and splenomegaly, affecting T-cell and B-cell numbers, altering the respiratory burst of macrophages, and changing NF-κB transcription factor activity and intracellular signaling [48]. For example, organophosphate pesticides can have direct or indirect immunotoxic effects. They can directly affect immune organs by inducing oxidative damage or altering the signaling pathways modulating their functions, resulting in the inhibition of serine hydrolases or esterases, which are crucial components of an effective immune system [117]. Indirectly, organophosphate pesticides can cause a dysregulation of the nervous system, or chronically alter the metabolism of immune organs [117]. Filipov et al. explored the effect the herbicide atrazine on mice. They reported a decrease in the weight of both the thymus and spleen along with a persistent decrease in the thymus cellularity [118]. In fact, exposure to atrazine leads to alterations in the cell phenotypes with an increase in CD8+ cytotoxic and memory T cells accompanied by a decrease in MHC-II+, CD19+ cells, and splenic naïve T helper cells [118]. Nevertheless, the results further showed that atrazine also inhibited dendritic cell maturation, as suggested by the decrease in the proportion of mature splenic dendritic cells (DC; CD11chigh) [118]. On the other hand, researchers were interested in the impact of enantioselectivity on increasing the toxicity of pesticides. Following exposure to an enantioselective acetofenate, an organochlorine insecticide, a macrophage-involved immunotoxicity was detected with strong effects on the generation of intracellular reactive oxygen species, induction of DNA damage, and upregulation of p53 gene expression [119].

Many organic solvents can also induce adverse immune effects and the requirement for their bioactivation to achieve immunotoxicity has been well established. Occupational exposure ranks first in the causes of solvent-associated toxicities [110]. Of these organic solvents, benzene and its metabolites have long been a topic of interest. In an experimental study, decreased levels of the complement system were observed in individuals that were occupationally exposed when compared to those that were not, showing the impairment of this immune system pathway [120]. A decrease in p53 gene expression, even at low exposure levels, was also reported in the exposed group, which confirms the carcinogenic effect of benzene [120]. Other studies have concluded that benzene metabolites can cause acute myeloid leukemia and possibly non-Hodgkin lymphoma, with two potential immunotoxic mechanisms. This is accomplished either by invasion caused by decreasing numbers of white blood cells, especially lymphocytes such as CD4+ T-cells, B-cells, and natural killer cells, while increasing proinflammatory cytokines, or by promoting carcinogenesis via impaired immunosurveillance [121]. In sum, benzene’s phenolic metabolites act together to produce the hematotoxic manifestations through DNA strand breaks, chromosomal damage, damage to mitotic spindle, sister chromatid exchange, and inhibition of topoisomerase II [122]. Whereas their immunotoxicity lies in inhibiting T dependent antibody responses, B- and T-cell lymphoproliferative responses, and cytotoxic T lymphocyte mediated tumor cell killing, and in rendering the host susceptible to infectious agents [110].

Halogenated aromatic hydrocarbons (HAHs) include the polychlorinated dibenzodioxins (PCDDs), polychlorinated dibenzofurans (PCDFs), and polychlorinated biphenols (PCBs). 2,3,7,8-Tetrachlorodibenzo-*p*-dioxin (TCDD), a member of PCDDs, is the most toxic halogenated aromatic hydrocarbon [110]. The toxicity of the latter has been discussed in a previous section. Experimental studies executed on animal models showed that in utero and lactational exposure to PCBs, PCDDs, and PCDFs are associated with persistent neurobehavioral, reproductive, and endocrine alterations [123]. On the other hand, polycyclic aromatic hydrocarbons (PAHs) such as benzo[*a*]pyrene (B[*a*]P) are metabolized to reactive metabolites that circulate to different tissues and covalently bind to DNA, RNA, and proteins altering normal cellular functions. White Jr. et al. evaluated the effect of 14 days of sub chronic exposure of ten polycyclic aromatic hydrocarbons in female B6C3F1 mice. The mice had significantly dampened antibody-forming cell responses and the immunosuppression occurred in a structure-related manner [124].

Nowadays, it is well known that workers chronically exposed to PAHs should regularly monitor their serum immunoglobulins. This is due to the marked depression of mean serum IgG, IgM, and IgA, and the tendency of serum IgE to reach higher values due to daily occupational exposure to PAHs [125]. Furthermore, the immunosuppressive synthetic methylated PAH, 7,12-dimethylbenz[*a*]anthracene (DMBA), has been proven to cause both an immediate and a sustained elevation of free intracellular calcium (Ca^2+^) in T cells [126]. By doing that, DMBA would be interfering with T cell activation following antigen binding to TCR, since it requires calcium mobilization. In fact, by depleting intracellular calcium stores, it would cause premature signaling, leading to tolerance [110]. As the PAH concentration gets higher, intracellular calcium also gets higher, culminating in apoptosis of lymphoid precursors in the bone marrow, circulating B and T lymphocytes, and lymphocytes in the thymus, spleen, and lymph nodes [110]. However, a protein tyrosine kinase (PTK) inhibitor, Genistein, has been shown to partially counteract PAHs’ rapid and sustained Ca^2+^ mobilization [126]. Bearing all this in mind, aromatic hydrocarbons are associated with immune suppression. The mechanisms will become more evident as this topic continues to be the focus of future research.

#### 4.2.4. Endocrine-Disrupting Chemicals

Each cell of the body is in one way, or another regulated by hormones, which execute their functions by acting on their respective cells [127]. Certain compounds known as endocrine-disrupting chemicals (EDCs) can interfere with an organism’s endocrine system and disrupt the immune activities, in a dose and structure related manner [48]. One can be exposed to EDCs, whether natural or human-made, through food, water, or through the air, and occasionally transdermally [127]. Bisphenol A (BPA) has been discussed as an example of EDCs’ immunotoxic mechanisms. When exposed to daily doses of BPA within the range of human exposure, animals had disrupted insulin secretion and glucose sensitivity as well as an accelerated postnatal growth. These findings provided a strong argument for the possible association between developmental exposure to EDCs, especially BPA, and the development of obesity later in life [128]. On a more immunological level, BPA can change the Th1/Th2 ratio by altering T-cell proliferation. Most studies concluded that the immune response becomes Th1 dominated with a decrease in Treg cells and an increase in macrophage production of TNF-α and nitric oxide [129]. Jefferson et al. were interested in phytoestrogen genistein (GEN), another EDC. They subjected mice to neonatal subcutaneous injections of genistein, which led to the development of multi-oocyte follicles. The results suggested that ovarian function and differentiation were disrupted in genistein treated mice with increasing severity over time [130]. They further proved that the fertility of these mice was reduced and if mating occurs, these effects can be transmitted to subsequent generations [130]. Actually, GEN acts as an agonist for estrogen receptor. It might be beneficial because of its anti-inflammatory role; however, its main effect is downregulating CD4+ T cells and the delayed type hypersensitivity response [48]. A study found that neonatal GEN exposure alters the growth of mammary glands as well as hormone receptor levels at all doses. Measurements of receptor levels showed increased levels of progesterone receptor protein and estrogen receptor mRNA in mice subjected to GEN compared to the control mice [131].

EDCs target various nuclear receptors such as the estrogen receptors α and β (ERα and ERβ), the androgen receptor (AR), the peroxisome proliferator activated receptors α and γ (PPARα, PPARγ), the pregnant X receptor (PXR), and the thyroid receptors α and β (TRα and TRβ) [132]. Recently, it has been shown that EDCs also interact with the retinoid X receptors (RXRα, RXRβ, and RXRγ), the estrogen related receptor γ (ERRγ), and the constitutive androstane receptor (CAR) [132]. Due to these interactions and different cross-links, EDCs can have various effects on the immune system. In reality, EDCs can act as both agonists and antagonists depending on the level of estrogen, which can shift the Th1/Th2 ratio accordingly. A high level of endogenous estrogen will cause a shift to a Th2 profile, which inhibits APC activation, T-cell proliferation, and pro-inflammatory cytokine secretion, whereas low or moderate levels of estrogen will shift the profile toward Th1, activating these mechanisms [48]. This was further evidenced in a study that described estrogen as a double-edged sword. Indeed, estrogen inhibits the production of TH1 proinflammatory cytokines such as IL-12, TNF-α, and IFN-γ, whereas they stimulate the production of TH2 anti-inflammatory cytokines such as IL-10, IL-4, and TGF-β [133]. This has caught the attention of several scientists since it explains the capability of estrogen to exacerbate or downregulate inflammatory diseases based on its level. Therefore, it is of no surprise that pregnant woman will have a Th2 dominated response because of their high estrogen levels and are more prone to Th2 mediated disease, whereas girls in their prepubescent years with low estrogen levels will be more prone to Th1 mediated diseases [48].

Similarly to estrogen, EDCs can also interfere with the synthesis of cytokines, immunoglobulins, and inflammatory mediators, and also shift the Th cell response [134]. For example, a lipopolysaccharide Th1 related chemokine, IFN-ɤ-inducible protein-10 (IP-10) and a Th2 related chemokine, macrophage-derived chemokine (MDC), were suppressed upon the administration of nonylphenol (NP) and 4-octylphenol (OP) [135]. This proposes the possibility that EDCs may suppress the Th1 response against intracellular pathogens and the Th2 response against bacterial and parasitic infections [135]. Another result proving this possibility is the dampening of TNF-α expression upon exposure to two EDCs, nitrofen and benzyl butyl phthalate, which led to the reduction of LPS-induced activation of macrophages, a key component in immunity against infections. Due to their ability to shift the Th response, EDCs influence allergic diseases and inflammation. EDCs may affect antigen-presenting cells (APCs) and subsequently direct Th2 polarization [134]. This mechanism was proposed after observing the effects of benzophenone, p-octylphenol, and tributyltin chloride, which decreased IL-12 while increasing IL-10 production by splenic APCs and shifting the immune response toward the Th2 end [136]. Furthermore, glutathione levels were largely reduced in APCs in EDC treated mice, ending up with exacerbation of airway inflammation [137]. Epidemiologic data also supported the hypothesis that some EDCs may have the ability to augment allergic diseases such as asthma, especially in children. In a pediatric cohort study, PVC flooring in the house, which contains phthalates, was shown to increase the risks of asthma and allergic rhinitis [138]. Similarly, diethyl-hexyl-phthalate in indoor dust is positively associated with wheezing at the preschool age [139]. The shift to a Th2 response promotes expression of Th2 cytokines, the most important being IL-4, and expanding the production of immunoglobulins, especially IgG1 and IgE [140]. For instance, BPA- and NP-treated mice [141] or OP-treated mice [142] showed increasing IL-4 production in a concentration-dependent manner. Likewise, two routinely used plasticizers, diethylhexylphthalate (DEHP) and di-isononyl phthalate (DINP), enhanced the activation of the IL4 gene promoter in T cells in mice [143]. On the other hand, EDCs may also affect the incidence and development of autoimmunity by interacting with infectious organisms [144]. When mice were subjected to organo-chlorine, a shortened disease-free time was observed, which confirms that EDCs accelerate the development of autoimmunity [145]. Similar results were observed when type 1 diabetes patients were exposed to EDCs, which accelerated the evolution of autoimmune thyroiditis [146]. Taking type 1 diabetes as an example of how EDCs affect diseases, studies compared the impact of different EDCs on this disease. BPA can worsen the progression of type 1 diabetes as seen from animal studies done by Bodin et al. They showed that BPA is able to decrease the numbers of macrophages in the pancreatic islets while augmenting the amount of Foxp3+ cells, resulting in an increased diabetic incidence [147], whereas GEN can decrease the incidence of this type of diabetes and protect from it by increasing insulin levels from β-cells, decreasing glucose production, and protecting from severe inflammation of pancreatic islets [148].

Some EDCs have also been found to alter the intestinal barrier function and disrupt the normal microbiota. They can be absorbed and transported to the liver, where they undergo conjugation before being excreted back into the gut through bile secretion for further microbial metabolism [149]. A cross-sectional study was interested in the passage of EDCs in the liver. They found that increases in markers for liver damage can be attributed to high levels of BPA. They further proved that higher BPA urinary concentrations were associated with increased prevalence of diabetes and cardiovascular disease [150]. Furthermore, when chemicals including EDCs are microbially metabolized, they can alter the normal structure, microbial genes, and molecules of the microbiota and lead to dysbiosis [151]. Dysbiosis is the result of the ability of EDCs to increase the barrier’s permeability, resulting in a leaky gut. This can be an open door to many diseases including type 1 diabetes, cardiac problems, and cancers [152]. Indeed, EDCs are associated principally with breast cancer development, but its association with colorectal cancer has recently become a booming subject of interest. This is because EDCs can potentiate inflammation and dysregulate the neuroendocrine-immune axis [152]. Numerous studies have used Caco-2 cells, which are derived from human colon carcinoma and are a model of inflamed IECs. When these cells were treated with GEN, decreased levels of IL-6 and MCP-1 were observed, suggesting a possible protective role in inflammation, which is an exception to previous studies on EDCs that showed that most EDCs aggravated the inflammatory pathways [48]. In fact, as previously discussed, EDCs have a major impact on immune cells and GALT’s immune cells are no exception. EDCs can alter GALT’s cytokine production and change the intestinal environment necessary for appropriate immune cells growth [48]. Finally, many factors come into play in the role of disease and immunological effects by EDCs including sex, diet, age of exposure, and structure/activity relationship of the EDC exposed [48]. Thus, further research needs to be done associating EDCs with these factors.

#### 4.2.5. Others

Cyclosporin A (CsA) downregulates the production of a variety of cytokines and inhibits the activation and maturation of many cell types involved in cell-mediated immunity. This immunosuppressive property enables it to be used as first line treatment in the rejection of organ transplants and many autoimmune diseases [153]. It has been shown that the powerful immunosuppressive drug cyclosporin A (CsA), inhibits the synthesis of certain T lymphocyte cytokines at the level of gene transcription [154]. In fact, Zipfel et al. demonstrated that CsA selectively inhibits IL-2, IL-4, and IFN-ɤ genes [155]. This is due to the ability of CsA to downregulate T-cell receptor mediated signal transduction pathways such as the inhibition of NF-ATc dephosphorylation by calcineurin and the blockade of PKC-activity [110]. Moreover, in a study done on larvae, cyclosporin A slightly suppressed lysozyme activity and markedly reduced antibacterial activity peptides against E. coli [156]. In addition to the direct effects on CsA on immunity, it also has indirect inhibitory effects on the growth and differentiation of B lymphocytes (IL-4 and IL-6), mononuclear phagocytes (IFN-γ), antigen-presenting cells (APCs), and natural killer (NK) cells [157]. Recent evidence also suggests its role in diminishing the mast cells’ production of mediators such as histamine and prostaglandin [157]. Study outcomes proved that renal allograft patients receiving CSA were not able to mount a cytotoxic response against EBV-infected B cells, which confirms the hypothesis that CsA dampens memory-T-cell proliferation, which contributes to a variety of diseases [158].

On the other hand, uncontrolled inflammation is the most common manifestation of several diseases. Glucocorticoids, which are released after IL-1, IL-6, and TNF-α stimulation, can antagonize the inflammatory effects while at the same time suppressing immunity. In reality, because of this function, they have been used for decades to treat allergies, reduce inflammation, and prevent rejection of transplanted organs [159]. Upon stimulation, glucocorticoids are rapidly secreted following a pattern of circadian and ultradian rhythms [160]. Following binding to their receptor, they influence the activity of major immune cells, dendritic cells, myeloid cells, and B- and T-lymphocytes by evoking apoptosis, altering differentiation and migration, and reducing cytokine release [161]. The newly formed glucocorticoid-receptor complex executes its actions through three mechanisms: glucocorticoid response elements binding to DNA; protein–protein interactions with other transcription factors; and several bindings to DNA and different transcription factors [160]. For example, Fowles et al. conducted a study on glucocorticoid-treated mallards. A 4 mg/kg dose reduced the hematocrit value but elevated alanine aminotransferase (ALT) activity, while significantly lowering their body weight [162]. However, the effects of glucocorticoids on the immune system are not always inhibitory. For example, the number of IgG Fc receptors on human mononuclear phagocytes induced by IFN-γ is dramatically enhanced [163]. Furthermore, glucocorticoid concentrations and IFN-γ play a key role in macrophage function. For instance, low glucocorticoid levels can boost, instead of inhibit, the ability of macrophages to ingest and attack infectious agents and autologous red blood cells [164].

Cyclophosphamide (Cy) is a strong anti-inflammatory drug capable of inducing immunosuppression by being both cytostatic and cytotoxic. It has been extensively used to treat cancers, transplant rejections, and autoimmune and inflammatory diseases [165]. Although used therapeutically, it can sometimes be toxic. Actually, for a number of immunosuppressive compounds including cyclophosphamide, their metabolic products rather than the parent compound are responsible for the toxicity [110]. Cyclophosphamide undergoes hepatic oxidation via P450 enzymes, then a series of enzymatic reactions to produce the reactive metabolites that have immunotoxic effects, of which acrolein is dominant [110]. Clinically, the latter can be associated with hemorrhagic cystitis, alopecia, and undesired immunosuppression. It can even cause damage to the heart when administered at high doses [166]. In an attempt to prove the impact of cyclophosphamide on the lungs, six patients were followed up. Results showed that the drug elicited pneumonitis in these patients; those with an early onset responded to discontinuation of the drug, whereas those with a late onset did not, and developed progressive pulmonary fibrosis on top of the pneumonitis [167]. Given its large impact on immunity, the researchers were interested in the mechanisms underlying cyclophosphamide’s immunosuppression. The latter led to a transient inhibition of delayed type hypersensitivity and depleted the lymphoid cells stores, causing lymphopenia, and markedly reduced macrophage activity [168]. Furthermore, 47 rheumatoid arthritis patients subjected to cyclophosphamide were compared to 22 not receiving any drug. The 47 patients had reduced numbers of circulating lymphocytes and had a depressed response to the nonspecific mitogen, phytohemagglutinin (PHA) [168]. Finally, data indicated that cyclophosphamide had more severe and long-lasting effects on the B cell compartment more than the T cell compartment [169].

Xenobiotics are chemicals to which the body is exposed that are extrinsic to the normal metabolism of the host [170]. Like other chemicals, xenobiotics can reach toxic concentrations. Xenobiotics inhibit, depending on the concentration, the nonspecific innate resistance of the organism, humoral, and cellular immune responses [171]. Xenobiotics can also disrupt the mechanism of programed cell death and subject the cells to an unwanted apoptosis, altering the number of immune-competent cells or lymphocytes, which may lead to serious adverse effects [172]. The major targets of xenobiotics are red cells, platelets, thyroid, kidney, and liver. Therefore, data showed that these drugs can cause autoimmune diseases, affecting these targets such as autoimmune hemolytic anemia (AHA) and autoimmune thrombocytopenia (ATP), autoimmune thyroiditis, autoimmune membranous glomerulonephropathy, and autoimmune liver disease [173]. The more a cell proliferates, the more it is a sensitive target for toxicants. This is the case of stem cells that are lost following xenobiotics treatment, resulting in myelotoxicity or bone marrow toxicity [1]. On the other hand, studies have been done on children exposed to xenobiotics throughout their gestational development, especially in the critical stages of hormonal, immunological, and neurological development. Outcomes have shown that this exposure had lifelong health and behavioral effects instead of being limited to birth defects [174]. Bearing all these effects in mind, studies were conducted with the aim of investigating the mechanisms underlying these immunotoxic changes. Results proved that there are four main sites at which xenobiotics might modify immune function. First, they can activate or injure immune cells and achieve a toxic tissue injury [175]. Second, a dysregulation of the immune function of lymphocytes might occur, potentially culminating in an autoimmune disease [175]. Third, xenobiotics can directly affect lymphocytes by influencing their maturation and differentiation [174]. Finally, they can stimulate approximately all the immune cells ranging from macrophages to granulocytes, platelets, and mast cells, and potentiate all biochemical systems and the secretion of lymphocytes’ cytokines [174]. However, detection of immune system alterations on exposure to xenobiotics is highly complicated, especially in humans because of various confounding factors such as age, sex, race, gender, co- existence of disease, diet, smoking… [176]. Thus, establishing a clear relationship between immunotoxicological data and risk prediction following xenobiotic exposure is still a challenge. Therefore, specific biologic substances might be used as markers of exposure to specific xenobiotics, which is the case of certain immunoglobulins. This is because xenobiotics can act as immunogens to stimulate the production of immunoglobulins as a part of an immune response [174].

Finally, chronic systemic inflammation characterized by immune-mediated or inflammatory states leads to endothelial dysfunction. One of the main players is (tumor necrosis factor) TNF-α, which increases the expression of adhesion molecules including intercellular adhesion molecule-1, vascular cell adhesion molecule-1, and E-selectin [177]. Reactive oxygen species may also contribute to the appearance of vascular lesions [177]. Furthermore, arginase’s expression is upregulated by TNF-α during ischemia/reperfusion injury. Once arginase is upregulated, L-arginine’s availability is decreased and reactive oxygen species such as O2^−^• is increased [178]. This subsequently leads to endothelial dysfunction. TNF-α inhibitors have been shown to decrease the atherosclerotic process. Vascular endothelial growth factor (VEGF), which is heavily involved in the atherosclerotic process, is also a main mediator of inflammation in diseases such as allergic rhinitis and systemic lupus erythematosus (SLE). Patients with SLE had the highest levels of VEGF. This provided evidence that serum VEGF levels depend on the type of immune response [179]. The levels are high in autoimmune diseases compared to Th2-polarized allergic reactions.

### 4.3. Psychological Stressors

Stress is the human’s body response to any kind of demand or threat. This response is characterized by the release of stress hormones including adrenaline and cortisol, which rouse the body for emergency action [180]. The heart and respiratory rates become faster, muscles contract, blood pressure increases, and senses become sharper [181]. Stress can have several vital individual physiological, psychological, and behavioral symptoms. Dysregulation of immune functions can occur through different pathways: the autonomic nervous system, the hypothalamic adrenal axis, extra-adrenal pathways involving neuropeptides, and neurotransmitters and neuroimmunological mediators [182]. In a study performed on chicken, they found that corticosterone increased the expression of the proinflammatory cytokines such as IL-1β, IL-6, and IL-18, messenger RNA levels of TGF-β4, and inflammatory ligands such as CCLi1, CCLi2, CCL5, CCL16, CXCLi1, and CXCLi2, which are potentially involved in modulating the adaptive immune response [183]. However, when administered chronically, corticosterone downregulated the proinflammatory cytokines, giving rise to the hypothesis that the delayed effects of chronic stress can suppress the immune response [183]. Thus, long-term stress is usually harmful, but short-term stress can be protective because it prepares the body to deal with threats [184]. It appears that the balance between Th1 and Th2/T regulatory cytokine production is altered in conditions associated with significant stress. Medical students were considered great candidates to study the effects of stress. Therefore, a study was conducted with them during stressful periods, and an increased incidence of type-2-mediated diseases such as viral infections, allergies, asthmatic reactions, and autoimmunity, was reported. This can be explained by the fact that stress is implicated in causing a shift in cytokine balance toward type 2, which leads to immune dysregulation [185]. However, Xiang et al.’s study yielded the opposite results. After 14 healthy volunteers were enrolled and subjected to an acute laboratory stressor, IFN-γ and IFN-γR mRNA levels increased while IL-10 levels decreased [186]. Therefore, they concluded that the acute stressor shifted the immune response toward Th1 cytokine and receptor production and that catecholamine-mediated signal pathways constitute a two-way communication with the central nervous system with stress playing a fundamental role in immune alterations [186]. On the other hand, other studies have focused on pregnant women. One of them explored the interplay between stress, fatigue, depression, and cytokines as markers of immune modulation, and found that stress was correlated with MIP-1*β*, which is a crucial cytokine in labor initiation [187]. Another study showed that higher prenatal stress affects both the innate and adaptive immune system. First, it altered the innate response by increasing the production of IL-8 and TNF-α after microbial stimuli. Second, upon dust mite stimulation, IL-13 production increased in the cord blood and reduced phytohemagglutinin-induced IFN-γ levels were reported, and by that altering the adaptive immunity [188]. The results of the experimental or clinical studies proved that stressors could alter the activities of immune cells in an intricate way that depends on the type of immune response, the physical and psychological characteristics of the stressor, and the timing of stress [189]. Age is another factor interfering with stress’s immunomodulatory effects. Data suggest that aging interacts with stress and depression to further lower the immune system’s capabilities, eventually enhancing the risks for morbidity and mortality among older adults [190]. Finally, stress is crucial and mandatory for the survival of the human body. However, as more studies are being published, one must learn to enhance the effects of “good” stress and reduce the effects of “bad” stress to maximally promote health and healing [184].

The environment also has an active role in promoting alterations to the immune system. Data suggest that industrialized countries have higher incidence of respiratory allergies [191]. Causative agents may be environmental gases, particles, passive smoking, and air pollution, which have been directly correlated with asthmatic diseases [191]. On the other hand, people of the upper class, with high socio-economic status, reported a higher frequency of allergic reactions, especially asthma symptoms. The difference in reporting between the upper and lower class could be attributed to the better awareness associated with better socio-economic situations [192]. Moreover, several characteristics related to the substance can affect the immune system. The route of exposure has a major impact on immunity. For instance, allergic contact dermatitis shows that topical exposure is correlated with a higher potential for sensitization [193]. However, an oral exposure renders the individual’s risk of tolerance higher, whereas the intravenous route is known to cause the most severe reactions of all routes [194,195]. Not only can the route affect the immune system, but also the regimen of exposure. For example, intermittent rifampicin treatment increased the frequency of immune-mediated tubulo-interstitial nephritis, which suggests that intermittent exposures relatively made sensitization easier and facilitated the development of immunoallergic reactions [196].

## 5. Methods for Assessing Immunotoxicity

Clinical pathology is utilized to assess immunotoxicity. By evaluating hematology, serum or plasma chemistry and urinalysis, we can assess the mechanisms, complementary responses, and consequences of immunotoxicity [197]. Evaluating the bone marrow can also help distinguish the primary effects from the secondary effects. A hypercellular bone marrow, along with an increased myeloid to erythroid (M/E) ratio in the setting of neutropenia could be caused by sepsis. In this setting, neutrophil production and release are both increased, but are unable to keep up with the demand. On the other hand, a hypocellular bone marrow with a decreased M/E ratio was seen with neutropenia due to direct suppression of production. In the former cases, sepsis caused neutropenia and in the latter, sepsis could result from neutropenia.

Hematological assessment includes measuring peripheral blood cell counts and differentials, bone marrow evaluation, reviewing slides for cell morphology, or the presence of a lift shift [198]. It is worth noting that blood drawn represents one time point and one compartment data. Pancytopenia refers to a decrease in all hematopoietic cells in circulation. Out of all cells, neutrophils have the shortest life span, around 10 h, and red blood cells have the longest, around 30 days. Since red blood cells have a longer lifespan, reticulocyte number is a more sensitive indicator of erythropoiesis than other parameters. White blood cells include lymphocytes, neutrophils, monocytes, basophils, and eosinophils. Concerning lymphocytes, the most common cause of lymphopenia or lymphocytosis is stress. Lymphopenia is also caused by corticosteroids and other drugs while lymphocytosis can be caused by recent vaccination, lymphoid neoplasia, diminished steroid production, or drug effects [199,200]. Table 2 describes the differences between stress and immunotoxicity in peripheral blood and serum chemistries. Different pieces of information are important in risk assessment. Bone marrow evaluation, along with flow cytometry, could be utilized to determine morphology, maturation progression, and M/E ratio [201].

Increased number of lymphocytes and plasma cells suggests immunostimulation or potential lymphoid neoplasms. Increased numbers of phagocytosed red blood cells usually indicate accelerated red blood cell destruction and suggests immunotoxicity due to antibodies directed against red blood cells.

Concerning serum chemistry, one should keep an eye on serum globulins, acute phase reactants, complement, and histamine. Some of the most common acute phase reactants are C-reactive protein and fibrinogen. The former works as an opsonin, facilitating phagocytosis, activating complement activation, inducing cytokine induction, and inhibiting chemotaxis and the neutriphil respiratory burst [202,203,204]. Fibrinogen helps in clot formation, facilitates chemotaxis, and enhances phagocyte function [204]. Serum amyloid-P and serum amyloid-A are also acute phase reactants heavily involved in the cascade of events. The former functions similar to C-reactive protein. The latter mainly inhibits chemotaxis of phagocytes and T cells, respiratory burst, lymphocyte proliferation, endothelial cell proliferation, and platelet aggregation. The remaining acute phase reactants that can be monitored are haptoglobin, ceruloplasmin, C3, α1-acid glycoprotein, α2 macroglobulin, α1-proteinase inhibitor (α1-antitrypsin), and α1-antichymotrypsin.

Recently, immunoinformatics has been the forefront of research areas such as allergy prediction to study immune-related genes, in silico vaccination, and T- and B-cell epitope prediction [205,206,207,208]. Toxicogenomics has been increasingly applied in immuntoxicity assessments [209]. Microarrays have been utilized in drug development to model pharmaceutical pharmacodynamic effects [210]. One example is seen in children with the TGF-β1-509TT genotype. Children carrying this genotype are at an increased risk of asthma when they are exposed to maternal smoking in utero or to traffic-related emissions [210,211]. Immunomics consists of the immune-related genomics and proteomics. In 2010, Diaz-Ramos et al. unraveled 1015 genes expressed in immune cells or lymphoid tissues linked to proteins on the plasma membrane [212]. Various immune-system related datatypes and databases exist. T-cell epitope databases including JenPep [213], the Syfpeithi database [214], FRED [215], IMGT [216], and IEDB 2.0 [217]. B-cell epitope databases include BCIPEP [218], CED [219], and Epitome [220]. Other databases are allergy prediction databases, databases related to the molecular evolution of immune genes and proteins [209].

## 6. Conclusions

A functioning immune system protects individuals from various stressors: chemical, physical, biological, and foreign substances along with tumor cells from within. This system requires a high level of coordination in order to interact with every antigen. Two types of immune responses exist: non-specific and acquired. The latter response could be further split into humoral and cell-mediated immunity. Since the immune system is intricate, it is vulnerable to the effects of toxic substances. An alteration in the immune response leads to either suppression, autoimmunity, or hypersensitivity. Many factors come into play when immunotoxicity is considered. Genetic factors, external risk factors, and the antigen involved are the three main pillars that dictate how the reaction proceeds. A lot of progress has been made over the past 40 years in immunotoxicology, and with the scientific advances, much more will be unraveled.

## Figures and Tables

**Table 1:** Function of T-Helper Cells.

	T-Helper 1 Cell	T-Helper 2 Cell	T-Helper 17 Cell	T-Regulatory
Secretes	IFN-γ, IL-2	IL-4, IL-5, IL-6, IL-10, IL-13	IL-17, IL-21, IL-22	TGF-β, IL-10, IL-35
Function	Activates macrophages and cytotoxic T cells to kill phagocytosed microbes	Activates eosinophils and promotes production of IgE for parasite defense	Immunity against extracellular microbes, through induction of neutrophilic inflammation	Prevents autoimmunity by maintaining tolerance to self-antigens
Induced by	IFN-γ, IL-12	IL-2, IL-4	TGF-β,IL-1, IL-6	TGF-β, IL-2
Inhibited by	IL-4, IL-10 (from T-helper 2 cell)	IFN-γ (from T-helper 1 cell)	IFN-γ, IL-4	IL-6

IFN-γ: interferon-gamma, IL: interleukin, Ig: immunoglobulin, TGF-β: transformation growth factor-beta.

**Table 2:** Differentiating between stress and immunotoxicity.

	Peripheral Blood	Serum Chemistry	Supportive Data
Stress leukogram: corticosteroid mediated	↓Lymphocytes, eosinophils ↑Neutrophils, monocytes Neutrophils are mature, may be hypersegmented	Hyperglycemia Lymphoid depletion in thymus Adrenal cortical hypertrophy	Overt organ toxicity Weight loss, anorexia Deaths in other animals in dose group. Findings only seen at doses at or higher than the maximum tolerated dose
Immunotoxicity: inflammation and infection	↑Neutrophils, monocytes ↓Lymphocytes (nonrodents) Immature neutrophils, toxic change	Mild nonregenerative anemia Inflammation in tissues Organisms Hypercellular bone marrow, increased M/E ratio	Clinical signs of infection, fever, anorexia, weight loss
